# Tissue-targeted R-spondin mimetics for liver regeneration

**DOI:** 10.1038/s41598-020-70912-3

**Published:** 2020-08-18

**Authors:** Zhengjian Zhang, Caroline Broderick, Marni Nishimoto, Teppei Yamaguchi, Sung-Jin Lee, Haili Zhang, Hui Chen, Mehaben Patel, Jay Ye, Alberto Ponce, Jennifer Brady, Hélène Baribault, Yang Li, Wen-Chen Yeh

**Affiliations:** Surrozen Inc., 171 Oyster Point Blvd, Suite 400, South San Francisco, CA 94080 USA

**Keywords:** Drug discovery, Chemical biology

## Abstract

R-spondin (RSPO) proteins amplify Wnt signaling and stimulate regeneration in a variety of tissues. To repair tissue in a tissue-specific manner, tissue-targeted RSPO mimetic molecules are desired. Here, we mutated RSPO (RSPO2 F105R/F109A) to eliminate LGR binding while preserving ZNRF3/RNF43 binding and targeted the mutated RSPO to a liver specific receptor, ASGR1. The resulting bi-specific molecule (αASGR1-RSPO2-RA) enhanced Wnt signaling effectively in vitro, and its activity was limited to ASGR1 expressing cells. Systemic administration of αASGR1-RSPO2-RA in mice specifically upregulated Wnt target genes and stimulated cell proliferation in liver but not intestine (which is more responsive to non-targeted RSPO2) in healthy mice, and improved liver function in diseased mice. These results not only suggest that a tissue-specific RSPO mimetic protein can stimulate regeneration in a cell-specific manner, but also provide a blueprint of how a tissue-specific molecule might be constructed for applications in a broader context.

## Introduction

The Wnt (“Wingless-related integration site” or “Wingless and Int-1” or “Wingless-Int”) signaling pathway plays a key role in the development, homeostasis and regeneration of many essential organs and tissues^1^. Timely activation, modulation, or enhancement of Wnt signaling holds potential for the treatment of various degenerative diseases and pathologies in which tissue regeneration could confer a therapeutic benefit.

R-spondins 1-4 (RSPO1-4) are a family of ligands that amplify Wnt signals through a receptor complex containing the zinc and ring finger 3 (ZNRF3) and ring finger protein 43 (RNF43) proteins and the coreceptor leucine-rich repeat-containing G-protein coupled receptors 4-6 (LGR4-6)^2–4^. RSPOs contain two furin (Fu) repeats, Fu1 and Fu2, which in combination are sufficient to recapitulate Wnt signaling enhancing activity^5^. Fu1 primarily interacts with ZNRF3/RNF43 and Fu2 interacts with LGR4-6^5–7^. ZNRF3 and RNF43 are membrane-bound E3 ligases that specifically target Wnt receptors (FZD1-10 and LRP5 or LRP6) for degradation^8,9^. Binding of RSPOs to ZNRF3/RNF43 and LGR4-6 causes clearance or sequestration of the ternary complex, which stabilizes Wnt receptors and amplifies Wnt signaling. In addition, R-spondins might work through mechanisms that are independent of LGRs^10,11^.

RSPOs may be beneficial in adult tissue repair^12,13^, particularly in situations where the expression of endogenous Wnt ligands is upregulated but signaling (presumably limited by receptor stability) is insufficient to overcome tissue damage. In liver, RSPO function is important for metabolic zonation and for hepatocyte proliferation and regeneration^12,14^. Therefore, RSPO may provide therapeutic benefit for various acute and chronic liver injury and diseases. One major challenge to exploring RSPO for tissue repair and regeneration is limiting RSPO effects to specific tissue of interest such as liver, as LGR4-6 and ZNRF3/RNF43 are widely expressed in various tissues. The mucosa of the gastrointestinal tract has high Wnt-dependent epithelial cell turnover, for example, and is known to be sensitive to exogenous RSPO treatment^15,16^. Strategies to avoid stimulation of the intestinal epithelium would be desirable for RSPO-mediated therapeutics targeting the liver.

To engineer liver-specific RSPO-like Wnt signaling enhancers, we embarked on a two-part strategy. First, we mutated RSPO in the Fu2 domain to specifically abolish the LGR interaction, rendering the molecule solely dependent on E3 ligases for activity. Second, we fused the mutant RSPO to single-chain variable fragments (scFv) or full-length IgG of an antigen-binding moiety targeting liver-specific receptors that potentially undergo rapid endocytosis. This coupling targets the Wnt enhancing activity of RSPOs to tissue-specific receptors, rather than to LGR. In mouse models, the engineered molecules specifically upregulated Wnt signaling and stimulated cell proliferation with greater tissue specificity than non-targeted counterparts, providing proof-of-concept for leveraging the regenerative properties of the Wnt signaling pathway in a potent, tissue-specific manner.

## Results

### LGR4/5/6 can be replaced by other cell surface receptors to facilitate RSPO-mediated Wnt signaling upregulation

**Figure 1:**
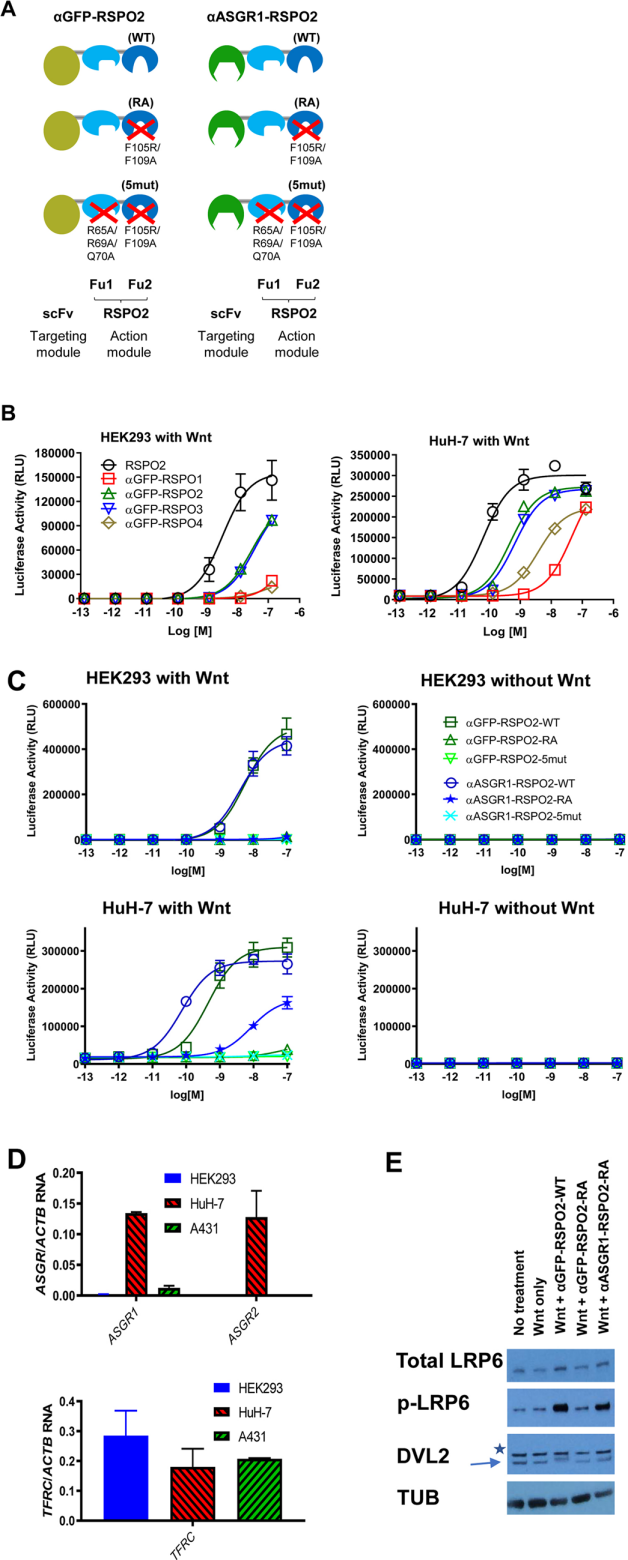
Design and characterization of cell type specific Wnt signaling enhancing mimetic molecules. (**A**) Scheme of designed molecules. Fragments of RSPO2 spanning the Fu1 and Fu2 domains (action module) were fused the C-terminus of cell type specific or control scFv (targeting module). Specific mutations used are indicated. (**B**) In vitro STF activity of four RSPO proteins. Furin domains of human RSPO1-4 were tested as fusions to an anti-GFP scFv. RSPO2 furin domain alone (N37-E143) was used as a control. Luciferase activity unit is arbitrary (RLU). (**C**) STF activity of RSPO mimetics on HEK293 (*top*) or HuH-7 (*bottom*) in the presence (*left*) or absence (*right*) of an exogenous Wnt source (30% Wnt3a conditioned media). (**D**) Quantitative PCR analysis of *ASGR1*, *ASGR2* and *TFRC* gene expression in HEK293, HuH-7 and A431 cells. The signals were relative to *ACTB*. (**E**) Western Blot analysis on LRP6 receptor and DVL2 phosphorylation in HuH-7 cells. Asterisk indicates a non-specific band that is detected in HuH-7 by the antibody used. The lower bands indicated by the arrow correspond to DVL2 with and without phosphorylation. Tubulin (TUB) is used as the loading control. 10% Wnt3a conditioned media was used as the exogenous Wnt source. Images were cropped for clarity. The uncropped images are presented in Fig. S2.

In this study, we investigated whether we could engineer a bi-specific RSPO by fusing an antibody that would bind to tissue-specific receptors to the module of RSPOs responsible for binding E3 ligases (i.e. negative regulators of Wnt signals). We introduced mutations in RSPO Fu2 domains to abolish LGR binding without perturbing with ZNRF3/RNF43 binding. Because LGR proteins are responsible for targeting RSPOs to a range of cell types^17^, these mutations would largely eliminate binding to non-specific tissues. The mutated RSPO was then fused to a scFv which binds a tissue-specific receptor. In this design, the intact Fu1 domain of the RSPO would bind the negative Wnt regulators, and the scFv would confer cell type/tissue specificity, as depicted in Fig. 1A. A full length RSPO protein contains thrombospondin (TSP) and basic region (BR) domains at C-terminus, in addition to Fu1 and Fu2 domains. TSP and BR domains, not furin domains, are implicated in LGR-independent signals^10^. Therefore, only the Fu1-Fu2 domains were used in this study.

In order to evaluate the potency and suitability for engineering of the four members of RSPO family, we first fused their Fu1-Fu2 domains with a scFv-containing the binding activity of an anti-GFP (αGFP) antibody. These mimetics were purified and used to treat the human embryonic kidney cell line (HEK293) and a human hepatocellular carcinoma cell line (HuH-7), which both expressed the Wnt-responsive SuperTop Flash (STF) luciferase reporter^18^. The N-terminal fusion of the αGFP scFv modestly reduced RSPO activity (compared to RSPO2 Fu1-Fu2 domains alone, Fig. 1B). However, all four αGFP-RSPO proteins showed robust stimulation of Wnt signaling in the presence of Wnt3a, with the RSPO2 and RSPO3 fusion proteins being the most potent in both reporter lines. RSPO2 and RSPO3 were therefore selected for further characterization.

To abolish LGR binding, we introduced point mutations at two highly conserved hydrophobic residues within the Fu2 domain of αGFP-RSPO2 (F105R and F109A) that are reported to be critical for binding to LGR proteins,^17^ (Fig. 1A, named αGFP-RSPO2-RA). To verify the involvement of the Fu1-E3 ligase interaction in the activity of the engineered molecules, point mutations reported to be critical for this interaction (R65A/R69A/Q70A)^5,7,19^ were also added (Fig. 1A, αGFP-RSPO2-5mut containing all five mutations, F105R/F109A/R65A/R69A/Q70A in Fu1 and Fu2). The impact of the Fu1 and Fu2 mutations on LGR and E3 ligase binding were verified using bio-layer interferometry (BLI). We measured the binding of wild type (αGFP-RSPO2-WT), or mutant (αGFP-RSPO2-RA or αGFP-RSPO2-5mut) proteins to recombinant LGR5 protein: wild type RSPO but not the two mutant RSPO variants exhibited binding to LGR5 (Fig. S1A). The effect of the Fu2 mutations was specific to the interactions with LGR, as binding of the αGFP-RSPO2-RA to E3 ligases (ZNRF3 and RNF43) was preserved (Fig. S1B). In contrast, mutations in both the Fu1 and Fu2 domains affected interactions with both the E3 ligases and the LGRs, as observed with αGFP-RSPO2-5mut (Fig. S1B). The impact of the Fu1 and Fu2 mutations on the ability of RSPO2 to enhance Wnt signaling was tested in HEK293 and HuH-7 STF cells. In the absence of any added Wnt, none of the RSPO2 derivatives was able to potentiate Wnt signaling (Fig. 1C right panels). In the presence of Wnt3a conditioned media, αGFP-RSPO2-WT enhanced Wnt3a activity in a dose-dependent manner. However, molecules with mutations in either Fu1 (αGFP-RSPO2-RA) or Fu1 and Fu2 (αGFP-RSPO2-5mut) were completely inactive, consistent with the expectation that native RSPO2 activity depends on its ability to engage both E3 ligases and LGR proteins.

To create a liver-specific RSPO-like Wnt signaling enhancer molecule, we chose to target the asialoglycoprotein receptor (ASGR), which is predominantly expressed on hepatocytes and undergoes rapid endocytosis^20^. ASGR is a hetero-oligomer composed of two polypeptides, ASGR1 and ASGR2. We converted a known ASGR1 binding antibody [^21^, WO2014/023709] into a scFv and fused it to the N-terminus of three RSPO2 variants to create αASGR1-RSPO2-WT, αASGR1-RSPO2-RA, and αASGR1-RSPO2-5mut fusion proteins (Fig. 1A right panel). Switching the targeting module from anti-GFP to anti-ASGR1 had no significant effect on the interaction of RSPO2 with E3 ligases or LGR5, while αASGR1-RSPO2-WT bound to both E3 ligases and LGR5. αASGR1-RSPO2-RA, however, lost the ability to interact with LGR5, and αASGR1-RSPO2-5mut lost the ability to interact with both LGR5 and E3 ligases (Fig. S1).

In order to determine the ability of the ASGR1-targeted RSPO2 mimetics to modulate cell type-specific Wnt signaling, we used a luciferase reporter assay to assess the activation of Wnt signaling in both HEK293 and HuH-7 STF reporter cells (Fig. 1C). In HEK293 cells, which do not express *ASGR1* (verified by quantitative PCR analysis, Fig. 1D), the αASGR1-RSPO2 fusion proteins behaved almost identically to their αGFP counterparts (Fig. 1C). In the absence of added Wnt, none of the αASGR1-RSPO2 proteins activated Wnt signaling (Fig. 1C right panel). In the presence of Wnt3a conditioned media in HEK293 cells, αASGR1-RSPO2-WT enhanced Wnt3a activity in a dose-dependent manner, like αGFP-RSPO2-WT, while both αASGR1-RSPO2-RA and αASGR1-RSPO2-5mut were inactive (Fig. 1C). In contrast, in HuH-7 cells which expresses *ASGR1* (verified by quantitative PCR analysis, Fig. 1D), αASGR1-RSPO2-RA significantly enhanced Wnt3a-induced signaling (Fig. 1C lower left panel) while αASGR1-RSPO2-5mut did not. Therefore, enhanced Wnt3a-induced signaling elicited by αASGR1-RSPO2-RA depends on its ability to bind E3 ligases. αASGR1-RSPO2-WT also showed a ~ sixfold increase in Wnt3a-induced signaling compared to αGFP-RSPO2-WT (Fig. 1C), suggesting that the attachment of the αASGR1 scFv may have synergized with LGR to further enhance the WT RSPO2 function.

In addition to the STF reporter assay, Western blots were used to examine Wnt signaling pathway components directly. As shown in Figs. 1E and S2, while the treatment of HuH-7 cells with Wnt3a conditioned media did not induce a significant change in phosphorylation of key proteins of the Wnt signaling pathway, treatment with αGFP-RSPO2-WT enhanced the Wnt3a-signalling and significantly increased levels of both phosphorylated LRP6 and DVL2 proteins, in addition to increasing total LRP6 protein levels. The mutations in Fu2 abolished any enhanced Wnt3a-signaling that was seen in the αGFP-RSPO2-RA treated cells. However, the fusion to αASGR scFv (αASGR1-RSPO2-RA) rescued the loss of function phenotype seen with αGFP-RSPO2-RA, slightly increased total LRP6 protein and phosphorylated DVL2 levels, and significantly increased levels of phosphorylated LRP6 (Figs. 1E and S2), suggesting that αASGR1-RSPO2-RA and RSPO2-WT amplified Wnt signaling to a similar extent.

### Validation of specificity by over expression of the targeted receptor

**Figure 2:**
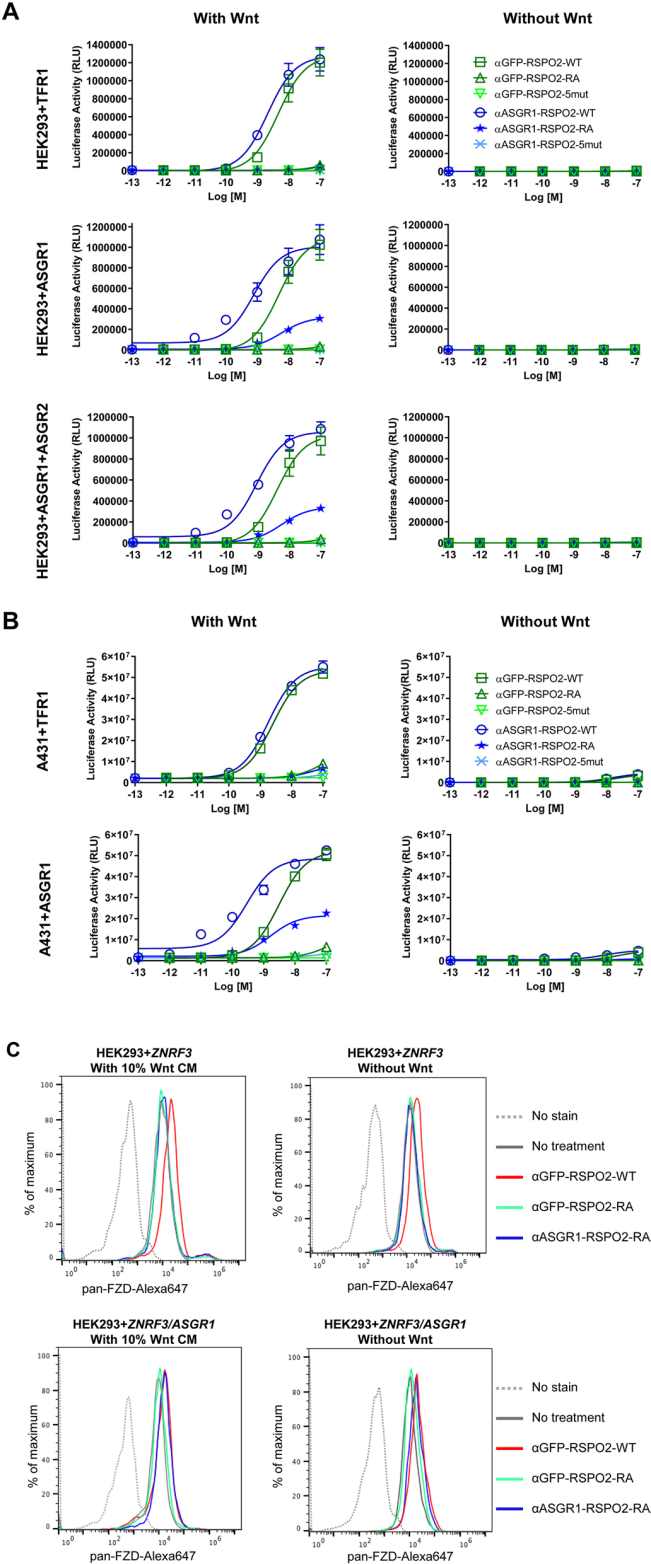
Confirmation of receptor-specific activation of Wnt signaling. (**A**) STF activity in HEK293 cells transiently transfected with *TFR1* (control, *top*), *ASGR1* (*middle*), and *ASGR1* together with *ASGR*2 cDNAs (*bottom*), either in the presence (*left*) or absence (*right*) of exogenous Wnt source (30% Wnt3a conditioned media). (**B**) STF activity in A431 cells with *TFR1* or *ASGR1* overexpression as specified. (**C**) Flow cytometry analysis of cell surface level of FZD proteins. HEK293 cells were transiently transfected with either *ZNRF3* alone (*top*) or *ASGR1* and *ZNRF3* cDNAs (*bottom*), treated with RSPO mimetics as specified at 10 nM, then stained by the pan-FZD antibody 18R5.

To further confirm that the cell-specific activity of α-ASGR1-RSPO2-RA was dependent on the presence of the targeted ASGR1, we transiently transfected HEK293 cells with a plasmid encoding a full length human *ASGR1* cDNA. Since ASGR1 and ASGR2 form a complex and neither are expressed in HEK293 cells (Fig. 1D), HEK293 cells were also co-transfected with both *ASGR* cDNAs. In addition, an unrelated receptor TFR1 (encoded by a *TFRC* cDNA) was transfected as a negative control. Like the un-transfected parental HEK293 cells, only the RSPO2-WT mimetics containing either αGFP or αASGR1 enhanced Wnt signaling in a Wnt-dependent manner in cells transfected with *TFRC* (compare Fig. 1C top panels to Fig. 2A top panels). In contrast, in cells transfected with either *ASGR1* alone or co-transfected with both *ASGR1* and *ASGR2*, the fusion of an α-ASGR1 scFv partially rescued the loss of function RSPO2-RA mutant’s ability to enhance Wnt signaling. *ASGR1* expression alone was sufficient to support the activity of αASGR1-RSPO2-RA, because the ASGR1 subunit can go through endocytosis independently of ASGR2^22^. The fusion of αASGR1 also increased the potency of RSPO2-WT protein, similar to that observed in HuH-7 cells (compare Fig. 1C lower panels to Fig. 2A lower panels). Activity of αASGR1-RSPO mimetics was entirely dependent on the presence of Wnt ligand (Fig. 2A right panels). The ASGR1-dependence of the αASGR1-RSPO mimetics was also confirmed in another ASGR1 negative cell line, A431, which is derived from a human epidermoid carcinoma and expresses *ASGR* genes at very low levels (Fig. 1D). Similar to what was observed in the HEK293 transfection studies, αASGR1-RSPO2-RA was only active in A431 cells expressing *ASGR1,* but not in A431 cells expressing the *TFRC* negative control receptor (Fig. 2B). Furthermore, the αASGR1 scFv also increased the potency of the RSPO2-WT fusion protein in an ASGR1-depedent manner, and the activity of all RSPO mimetics were completely dependent on the presence of Wnt ligand (Fig. 2B right panels). Collectively, these results demonstrated that RSPO-mediated downregulation of E3 ligases through binding of LGR proteins can be replaced by targeting RSPO to a different cell surface receptor, ASGR1. The resulting RSPO mimetics fully resembled WT RSPO in Wnt dependency and actions through E3 ligases but differed in that Wnt signaling enhancement was cell type specific.

To further validate the mechanism of action of the RSPO mimetics, we examined FZD receptor levels on the cell surface. To sensitize the system, we overexpressed *ZNRF3* by transient transfection before treatment. We used the pan-FZD antibody 18R5^23^ to stain the cells, and found that αGFP-RSPO2-WT clearly increased the signal of FZD surface levels when compared to the untreated cells, while no increase was observed with αGFP-RSPO2-RA or αASGR1-RSPO2-RA treatment (Fig. 2C top). This is consistent with the lack of activity observed with these two RSPO-RA constructs in HEK293 cells. In contrast, when cells were co-transfected with both *ZNRF3* and *ASGR1*, αASGR1-RSPO2-RA increased pan-FZD staining similar to αGFP-RSPO2-WT, while αGFP-RSPO2-RA did not (Fig. 2C bottom). Therefore, the ability of αASGR1-RSPO2-RA to stabilize cell surface levels of FZD was dependent on the presence of ASGR1 and correlated well with the Wnt signal-enhancing activity of the ASGR1-targeted RSPO mimetic molecule.

### Validating the RSPO mimetic concept with a second cell surface receptor and RSPO3

**Figure 3:**
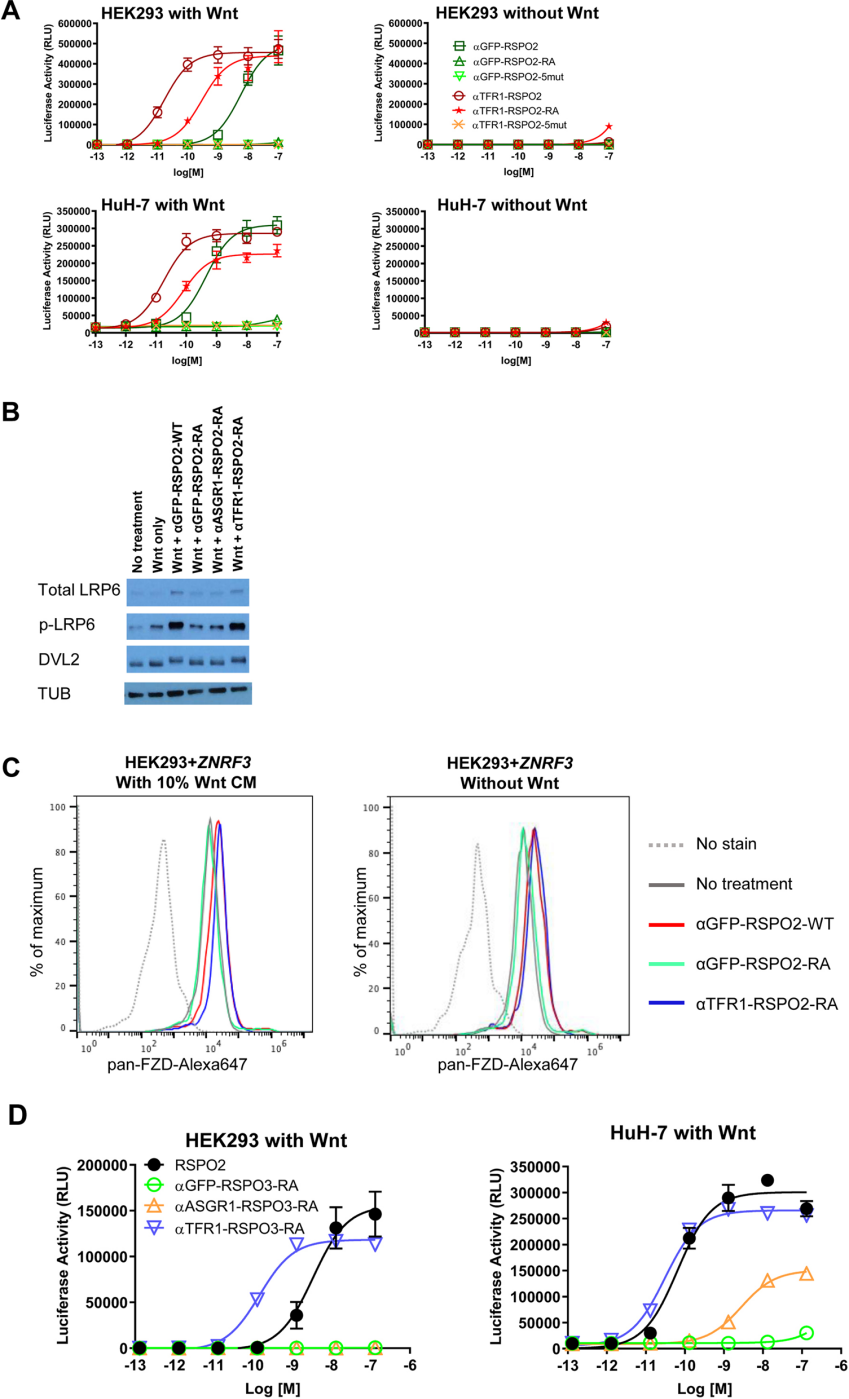
Application of the targeted RSPO mimetic design to a transferrin receptor and RSPO3. (**A**) STF activity of specified proteins on HEK293 (*top*) or HuH-7 (*bottom*) in the presence (*left*) or absence (*right*) of exogenous Wnt source (30% Wnt3a conditioned media). (**B**) Western Blot analysis for LRP6 receptor expression and DVL2 phosphorylation in HEK293 cells. Tubulin (TUB) is used as the loading control. Images were cropped for clarity. The uncropped images are presented in Fig. S2. (**C**) Flow cytometry analysis of cell surface level of FZD proteins. (**D**) STF activity of RSPO3-based mimetic molecules in HEK293 (left) and HuH-7 (right) cells, in the presence of 30% Wnt3a conditioned media.

As a further proof-of-concept to our design of liver-specific RSPO mimetic molecule, we tested a second bi-specific construct targeting a different cell surface receptor, the transferrin receptor 1 (TFR1). Like ASGR1, TFR1 has been reported to undergo continuous endocytosis^24^. However, while ASGR1 expression is hepatocyte specific, TFR1 is broadly expressed in many cell types. We constructed and characterized fusion proteins containing a scFv binder to human TFR1 [^25^, WO2016/081640] with RSPO2-WT, RSPO2-RA, or RSPO2-5mut. Similar to the anti-GFP or anti-ASGR1 fusion proteins, the TFR1 targeted RSPO2-RA (αTFR1-RSPO2-RA) mimetic lost the ability to interact with LGR5, and the TFR1 targeted RSPO2-5mt (αTFR1-RSPO2-5mut) lost the ability to interact with both LGR5 and E3 ligases (Fig. S3). When tested in the HEK293 STF reporter assay in the presence of Wnt, αTFR1-RSPO2-RA stimulated Wnt signaling while αGFP-RSPO2-RA did not (Fig. 3A). Interestingly, αTFR1-RSPO2-WT showed a 20-fold enhancement compared to αGFP-RSPO2-WT. Similar results were obtained in HuH-7 STF cells, consistent with the expression of the targeted receptor in both cell lines (Fig. 1D). Consistent with the mechanism of RSPOs, no Wnt signaling activity was observed in the absence of Wnt ligand (Fig. 3A right panels). The increased potency observed with anti-TFR1 RSPO2 fusion proteins highlights the tractability of using engineered molecules for therapeutic benefit: the right combination of receptor and scFv binding element allows tuning of the magnitude of Wnt response and achieving the optimal activity for RSPO mimetic molecules in a cell type-specific manner. Factors contributing to magnitude of activity may include the abundance of the targeted receptor, exposure of epitopes, kinetics of binding and dissociation of the targeting module and the cycling of the receptors etc. As was seen with αGFP-RSPO2-WT and αTFR1-RSPO2-RA, but not αASGR1-RSPO2-RA, there was increased phosphorylation of LRP6 and DVL2 and increased total LRP6 protein levels in the presence of Wnt, in HEK293 cells (Figs. 3B and S2). The effects of the TFR1-targeted RSPO mimetics on cell surface FZD receptor levels was also examined by flow cytometry. Treatment of HEK293 cells with αGFP-RSPO2-WT and αTFR1-RSPO2-RA, but not αGFP-RSPO2-RA, increased the FZD receptor levels on these cells measured by a pan-FZD antibody (Fig. 3C).

Next, we examined whether similar strategies could be used to generate RSPO mimetic molecules with other RSPO family members. We utilized RSPO3 to generate various fusion proteins for its potency in the STF assay (Fig. 1B). Residues F106 and F110 in RSPO3 correspond to the two hydrophobic residues for LGR binding domain in RSPO2, F105 and F109^17^. Consistent with the strategy for RSPO2 fusion proteins shown in Fig. 1A, we generated αGFP-RSPO3-RA, αASGR1-RSPO3-RA, and αTFR1-RSPO3-RA. As expected, a non-targeted fusion protein comprising of RSPO3 with F106R/F110A mutations (αGFP-RSPO3-RA) did not enhance Wnt signaling in HEK293 STF cells (Fig. 3D). Since HEK293 cells express TFR1 receptor, but not ASGR1 receptor (Fig. 1D), αTFR1-RSPO3-RA was able to confer Wnt signaling activity, but αASGR1-RSPO3-RA remained inactive. In HuH-7 STF cells, where both ASGR1 and TFR1 receptors are expressed (Fig. 1D), αASGR1-RSPO3-RA and αTFR1-RSPO3-RA were both able to restore activity of the RSPO3-RA mutant (Fig. 3D). Similar to the RSPO2 mimetics (Figs. 2 and 3), αTFR1-RSPO3-RA was more potent than αASGR1-RSPO3-RA. Similar to the observation with RSPO2-based constructs, this observation is presumably due to increased cell surface receptor expression, and/or more favorable receptor internalization kinetics, etc.

### Systemic delivery of αASGR1-RSPO2-RA induces Wnt target gene expression in liver while avoiding small intestine

**Figure 4:**
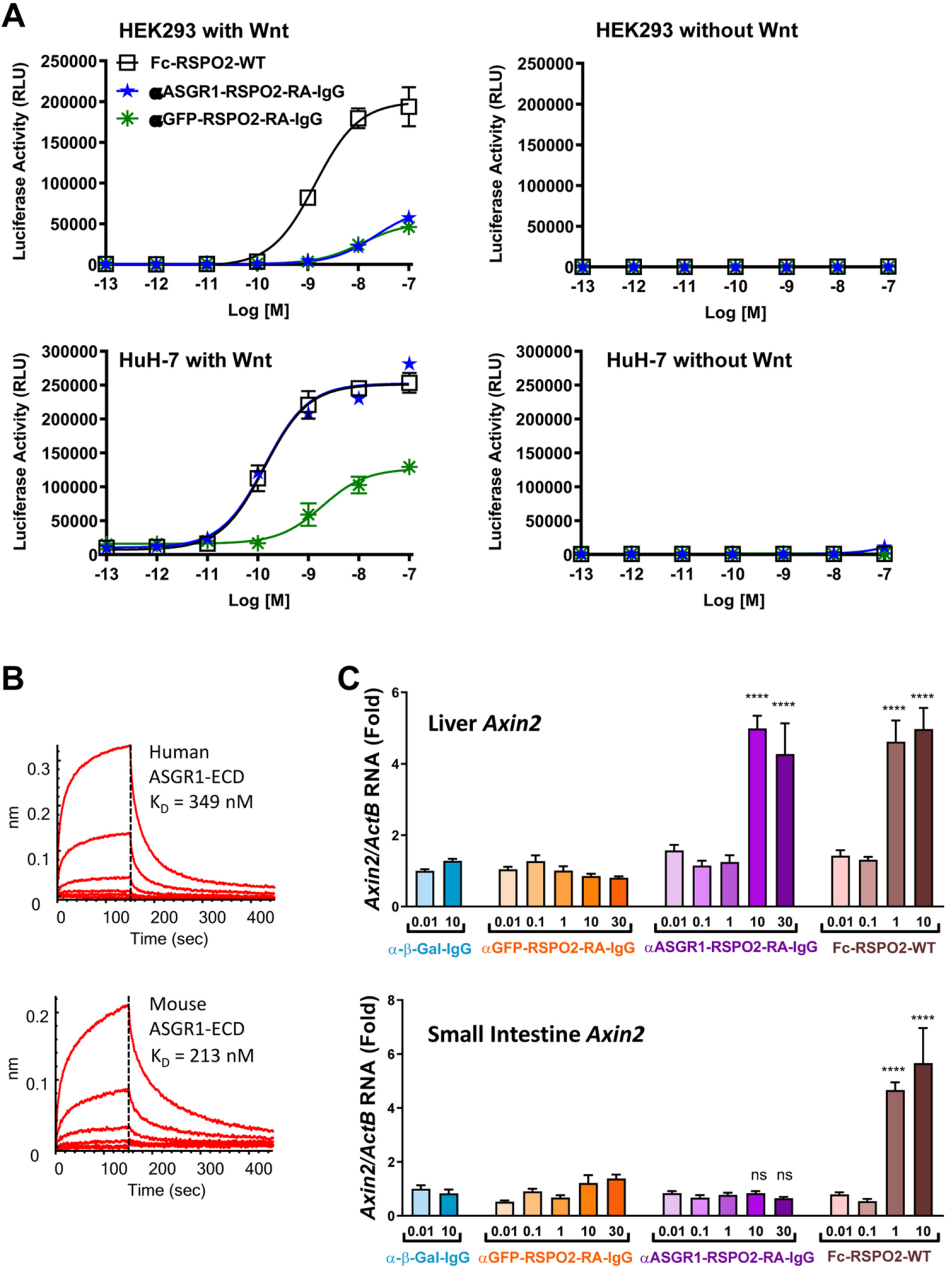

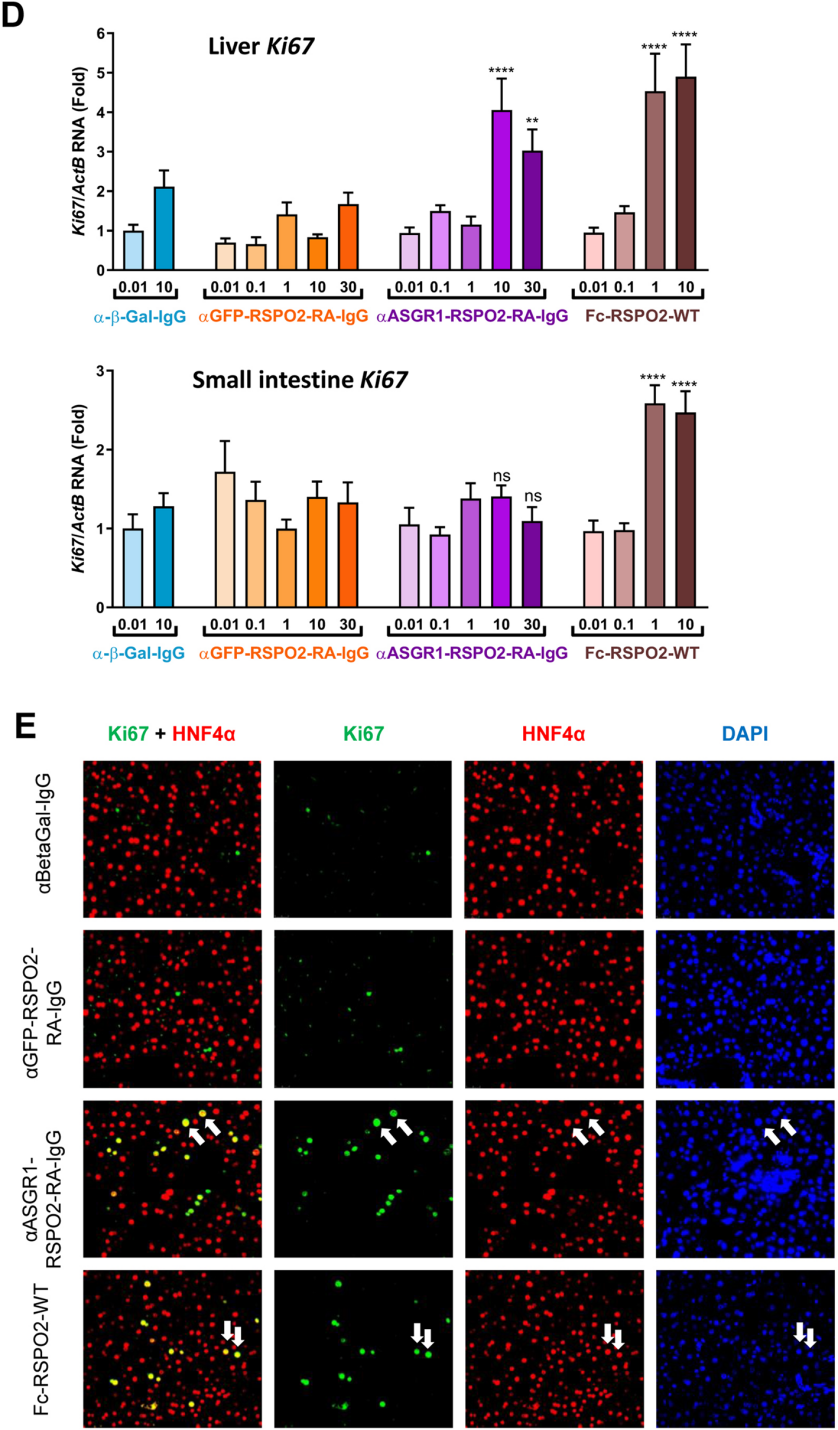
In vivo tissue-specific enhancement of Wnt signaling. (**A**) STF activity of the RSPO mimetic molecules in the appended-IgG format. (**B**) BLI analysis of the ASGR1 antibody (expressed as a Fab) on human and mouse ASGR1. The affinity (Kd) determined by steady-state fitting is indicated. (**C**) *Axin2* and (**D**) *Ki67* expression in liver (*upper panel*) and small intestine (*lower panel*) were analyzed by quantitative PCR, 48 h after i.p. injection of proteins as specified (n = 8 mice per group). Statistical analysis was performed using 1-way ANOVA: (ns) not significant, **p < 0.01, ****p < 0.0001. (**E**) Immunofluorescence staining of liver samples for Ki67 and HNF4α from 10 mg/kg treatment groups. White arrows denote some double-positive cells as examples. DAPI was used to stain nuclei.

To determine whether αASGR1-RSPO2-RA can activate the Wnt signaling pathway in a tissue-specific manner in vivo, mice were treated with αASGR1-RSPO2-RA and control proteins. We have engineered an αASGR1-RSPO2-RA protein with predicted prolonged half-life in vivo by switching the scFv of the binders to an IgG format, where the RSPO2 is fused to the N-terminus of the IgG heavy chain. In vitro reporter assays showed αASGR1-RSPO2-RA in IgG format (αASGR1-RSPO2-RA-IgG) to be active and cell type-specific compared to wild type RSPO2-IgG (Fig. 4A). αASGR1-RSPO2-RA showed higher activity in vitro in the IgG format than the scFv format, though it also had higher background activity as revealed by αGFP-RSPO2-RA-IgG. These activity increases might be due to increased avidity from the bivalency of the appended-IgG format as compared to the scFv format. Interestingly, bivalent antibodies against ZNRF3 have been reported to show mild RSPO-like activities in vitro^9^.

In order to determine whether these RSPO mimetics demonstrate utility/specificity in a biologically relevant context, we chose to examine the activities of the ASGR1-targeted molecule and control molecules in IgG format in vivo. The αASGR1 antibody used in these studies has similar affinity to both the human and murine ASGR1 (Fig. 4B).

First, we performed a dose response of αASGR1-RSPO2-RA-IgG compared with αGFP-RSPO2-RA-IgG and other controls in naïve mice to examine induction of Wnt target gene *Axin2*. Mice received intraperitoneal (i.p.) injections of tested proteins, ranging from 0.1 to 30 mg/kg. Liver and small intestine samples were collected 48 h (h) later for tissue phenotyping and gene expression analyses. Fc-RSPO2-WT increased *Axin2* expression at 1 and 10 mg/kg treatment in both liver and small intestine (Fig. 4C), consistent with previous reports^12,15^. In contrast, αASGR1-RSPO2-RA-IgG at 10 and 30 mg/kg induced *Axin2* expression in liver, but not in small intestine. Importantly, this specificity is dependent on the αASGR1 targeting module, because the untargeted molecule αGFP-RSPO2-RA-IgG showed no effect in vivo on *Axin2* expression in either tissue up to 30 mg/kg treatment (Fig. 4C). No effect on *Axin2* expression was observed with control antibody anti-β-galactosidase (α-β-Gal-IgG) at 0.01 and 10 mg/kg, further confirming specificity of the targeted molecules.

Similar tissue specificity of αASGR1-RSPO2-RA-IgG and dependency on the targeting module was also observed in expression of a proliferation marker, *Ki67* (Fig. 4D). Consistent with quantitative PCR analysis, an increase in crypt length and abundance of Ki67 positive cells in the small intestine was observed by histological analysis and immunofluorescence respectively in response to Fc-RSPO-WT, but not to αASGR1-RSPO2-RA-IgG (Fig. S4A). To examine whether or not proliferation occurred in hepatocytes, we co-stained liver samples with antibodies specific for Ki67 and for the mature hepatocyte marker HNF4α. Livers treated with αASGR1-RSPO2-RA-IgG and Fc-RSPO2-WT appeared to have increased numbers of Ki67 positive nuclei that co-localized with HNF4α, while livers treated with αGFP-RSPO2-RA-IgG or α-β-Gal-IgG appeared to have fewer Ki67 positive nuclei (Fig. 4E). No histological differences were observed in the liver of the different groups (Fig. S4B). Taken together, these results suggest that αASGR1-RSPO2-RA-IgG can activate the Wnt pathway in a liver-specific manner in vivo, that αASGR1-RSPO2-RA-IgG-induced increases in Wnt signaling activity is sufficient to stimulate proliferation, and that hepatocytes are the primary cell population responsive to this stimulation.

### Liver-targeted RSPO mimetics improves liver function in diseased mouse models

**Figure 5:**
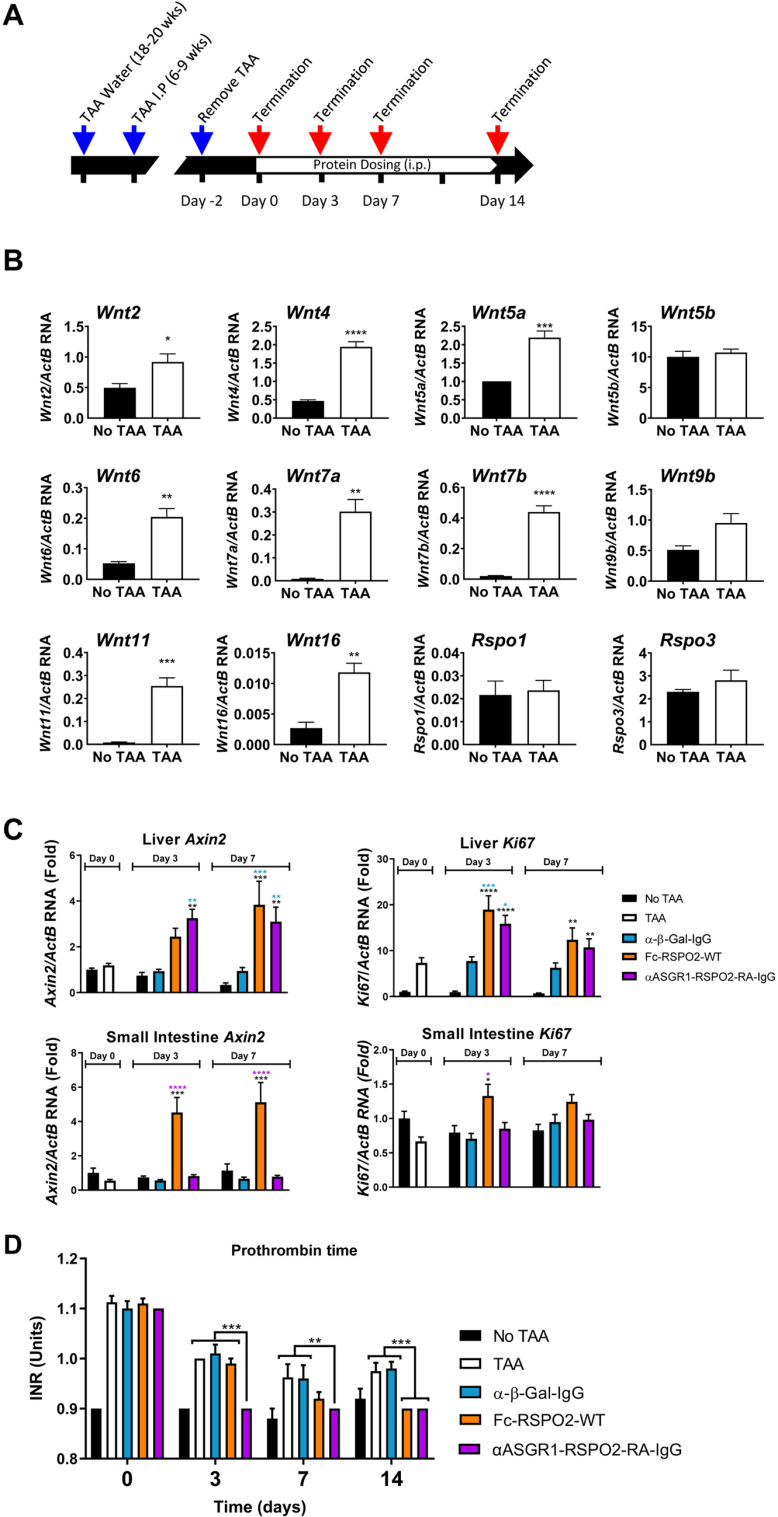
Effects of liver targeted RSPO mimetic in a TAA mouse model of chronic liver disease. (**A**) Study design. (**B**) Quantitative PCR analysis of *Wnt* and *Rspo* RNA in the livers of normal and TAA treated mice. Genes below detection limit were not shown. (**C**) Quantitative PCR analysis of *Axin2* (*left*) and *Ki67* (*right*) RNA in liver (*top*) and small intestine (bottom; n = 10 mice per group). (**D**) INR of prothrombin time during treatment. Statistical analysis was performed using 1-way ANOVA: *p < 0.05, **p < 0.01, ***p < 0.001, ****p < 0.0001 (n = 10 mice per group).

We next examined the activity and efficacy of αASGR1-RSPO2-RA-IgG in a mouse model of liver fibrosis (Fig. 5A). We utilized a chronic thioacetamide (TAA) model, in which extensive liver fibrosis was observed and liver function was impaired^26^. We found that upon damage by TAA treatment, Wnt signaling pathway components, including genes encoding many Wnt ligands were upregulated in liver (Fig. 5B). In contrast, expression of the *Rspo1* and *Rspo3* remained unchanged (*Rspo2* and *Rspo4* were below detection limit) (Fig. 5B and data not shown). Therefore, we tested whether administration of liver specific RSPO mimetics could confer therapeutic benefit in this context.

The experimental design regarding duration of TAA pre-treatment and subsequent protein treatment (αASGR1-RSPO2-RA-IgG and control proteins) is outlined in Fig. 5A. We conducted two studies of similar design. In one study, mice were terminated at 0-, 3- and 7-days following initiation of protein treatment. In a second study, mice were terminated at 14 days after initiation of protein treatment. Liver and small intestine samples were collected and assessed for changes in gene expression. A panel of Wnt-target genes were analyzed and found to be induced by Fc-RSPO-WT and αASGR1-RSPO2-RA-IgG in the liver (Fig. S5A). The liver plays an important function in the detoxification of circulating toxins via cytochrome P450 enzymes. Two of these enzymes, *Cyp1a2* and *Cyp2e1*, are direct Wnt-target genes and are elevated after treatment with αASGR1-RSPO2-RA-IgG or Fc-RSPO2-WT, suggesting a potential benefit for a liver-specific RSPO mimetic in this liver function (Fig. S5A). However, αASGR1-RSPO2-RA-IgG led to a liver-specific increase of *Axin2* expression at days 3 and 7, while Fc-RSPO-WT induced *Axin2* in both liver and small intestine (Fig. 5C). No change was observed with the α-β-Gal-IgG control treatment. αASGR1-RSPO2-RA-IgG also increased *Ki67* expression in a liver-specific manner (Fig. 5C). Consistent with these findings, an increase in crypt length and abundance of Ki67 positive cells in the small intestine was observed by histological analysis and immunofluorescence respectively in response to Fc-RSPO-WT, but not to αASGR1-RSPO2-RA-IgG (Fig. S5B).

To monitor liver function, blood samples were taken at indicated times during treatment and used to measure International Normalized Ratio (INR) of prothrombin time and total bilirubin. We have found that normal mice have an INR of approximately 0.9, since the values are normalized to standard human prothrombin time that is longer than mice. Prothrombin time measures function of clotting factors which are made in liver. In severe liver diseases, insufficient synthesis of clotting factors leads to prolongation of prothrombin time (increased INR). TAA treatment increased INR to 1.1 due to serious liver damage (Fig. 5D). Upon TAA removal, mice are known to exhibit some natural resolution of liver damage, consistent with the INR drop observed in untreated animals from 1.1 at day 0 to < 1.0 at day 14. However, αASGR1-RSPO2-RA-IgG accelerated INR recovery, as 100% of αASGR1-RSPO2-RA-IgG treated mice had an INR of 0.9 by day 3. Fc-RSPO2-WT treated mice had reduced INR compared to the α-β-Gal treated mice at day 7 and reached the same effect as αASGR1-RSPO2-RA-IgG at day 14. Therefore, the liver-specific αASGR1-RSPO2-RA-IgG molecule can improve liver synthetic function in chronic liver disease model. A reduction in serum total bilirubin and a trend in higher liver AFP expression are also observed at termination in response to αASGR1-RSPO2-RA-IgG (Fig. S5A).

Histological analysis of these samples at day 14 revealed the presence of oval cells, infiltration of monocytes and bridging fibrosis in all groups (Fig. S5C). There were no significant differences in these histological features regardless of the test articles administered over 14 days.

## Discussion

By amplifying endogenous Wnt signaling, RSPOs are important factors for tissue regeneration. One major limitation to using RSPOs for tissue repair and regeneration is the lack of tissue specificity. To harness the regenerative potential of RSPOs in a tissue- or cell-specific manner and to further understand the biology of RSPOs, we engineered tissue-specific RSPO mimetics (e.g. αASGR1-RSPO2-RA) by redirecting the LGR reliance of RSPO to a tissue-specific cell surface receptor. Evidence supporting successful generation of functional, tunable, tissue specific RSPO mimetic molecules derives from the following observations. First, these bi-specific RSPO mimetics did not exhibit LGR binding and lost the ability to potentiate Wnt signals in various LGR-expressing cells, while retaining their ability to bind E3 ligases. Second, the targeted RSPO mimetic molecules are dependent on target receptor expression to function like RSPOs in potentiating Wnt signals in the presence of Wnt. Third, the mechanism of our bi-specific RSPO mimetic molecules appears similar to native RSPOs in increasing cell surface expression of FZDs and enhancing signaling through phosphorylation of LRP6 and DVL. Finally, our targeted, bi-specific αASGR1-RSPO2-RA specifically activated Wnt signaling in vivo and enhanced cell proliferation in liver (specifically in hepatocytes) but not in other tissues, such as the intestine, which is more sensitive than the liver to treatment with wild type RSPO proteins^15,16^. This cell type specific RSPO mimetic concept is also validated by a recent report where E3 ligase binding antibody fragments were fused to cytokine IL-2 and directed RSPO like response to cells expressing IL-2 receptor, CD25^27^.

Recently, LGR-independent mechanisms have been reported for RSPO2 and RSPO3 function^10,11^, but precise mechanisms remain to be elucidated. One potential mechanism seems to be mediated by the TSP type 1 domain and the BR of RSPO by interacting with heparan sulfate proteoglycans (HSPGs) which are broadly distributed on the cell surface^10^. By including only the furin domains, our RSPO mimetic is, by design, independent of the TSP and BR domains. Our study of these functional RSPO mimetic proteins provides independent evidence that RSPO2 and RSPO3 can function independent of LGR4/5/6, provided that the mutated RSPO2 or RSPO3 is targeted to specific cell surface receptors.

TAA administration in mice is a widely used model of liver cirrhosis^26^. It induces bridging fibrosis similarly observed in human liver biopsies of patients, a mild but consistent elevation in total bilirubin, and an increase in prothrombin time, or its derivative, the INR, since the liver synthetizes clotting factors. We demonstrate that treatment with a liver-targeted R-spondin mimetic improved liver functions in a TAA model, supporting its potential for therapeutic development. The TAA model in rats has been used to reveal the anti-fibrotic effect of some agents after 6 to 8 weeks of treatment. Longer periods of αASGR1-RSPO2-RA treatment may be needed to determine whether increases in hepatocyte proliferation observed here will have an impact on fibrosis.

In addition to ASGR1 liver specific RSPO mimetic, we have also successfully applied this strategy to design molecules specifically enhancing Wnt signaling in oral mucosa (data not shown). Future investigations will further delineate whether the RSPO mimetics and RSPO biology work strictly through endocytosis of E3 ligases or through additional mechanisms. This approach opens new opportunities for the development of Wnt-based therapeutics for regenerative medicine. Further studies of these RSPO mimetics will improve our understanding of both RSPO biology and the utility of RSPO mimetics for targeting disease treatment or repair to specific tissues.

## Material and methods

### Molecular cloning

The anti-ASGR1 scFv was based on reported sequence from published patent application WO 2014/023709 A1. The anti-TFR1 scFv was based on published patent application WO2016/081640 A1 (clone 7A4). The anti-GFP scFv was from an in-house screening (Aaron Sato, unpublished). The anti-β-galactosidase IgG (α-β-Gal-IgG) used for in vivo studies was constructed based on reported sequences^28^, using the human IgG1 LALA-PG backbone^29^. Fc-RSPO2-WT was constructed by fusing RSPO2 (S36-E143) to the C-terminus of human IgG1 (LALA-PG) via the 15-mer linker. A cleavable version was made by inserting an HRV 3C cleavage site (LEVLFQGP) right before the RSPO2 sequence to generate monovalent RSPO2 protein. More details regarding molecular cloning are available upon request.

### Protein production

All recombinant proteins were produced in Expi293F cells (Thermo Fisher Scientific, Waltham, MA) by transient transfection unless otherwise specified. All scFv-containing fusions and the anti-ASGR1 Fab were first purified using complete his-tag purification resin (Sigma-Aldrich, St Louis, MO) following vendor recommended procedures. All IgG-based and Fc-containing constructs were first purified with Protein-A resin and eluted with 0.1 M glycine pH 3.5. All proteins were then polished by a size exclusion column in HBS buffer (10 mM HEPES pH 7.2, 150 mM NaCl). Proteins were supplemented with glycerol to 10% for long term storage at −80 °C. In vitro biotinylated receptors were carried out as previously described^13^, followed by further purification by size exclusion chromatography. All proteins tested were examined by SDS–polyacrylamide electrophoresis and estimated to be at least 90% pure.

### SuperTop Flash (STF) assay

Wnt signaling activity was measured using cell lines containing a luciferase gene controlled by a Wnt-responsive promoter (STF reporter) as previously reported^18^. In brief, cells were seeded at a density of 10,000 per well in 96-well plates 24 h prior to treatment, then treated with wild type RSPO or mimetic proteins overnight, either alone or together with 30% Wnt3a-conditioned media. Wnt3a-conditioned media was prepared from ATCC-CRL-2647 Wnt3a-secreting L cells following vendor recommended conditions. Cells were lysed with Luciferase Cell Culture Lysis Reagent (Promega, Madison, WI) and activity was measured with Luciferase Assay System (Promega, Madison, WI) using vendor suggested procedures. Data were plotted as average −/+ standard deviation of triplicates and fitted by non-linear regression using Prism (GraphPad Software, San Diego, CA). For overexpression of exogenous receptors, cells were transiently transfected with plasmids containing receptors of interest under eukaryotic expression promoters (*ASGR1* was clone OHU03658D from GenScript, *ASGR2* was in-house cloned isoform d/NP_001188281.1, and *TFRC* was clone HG11020-UT from SinoBiologicals), then split into 96-well plates (20,000 cells per well) for STF assay 24 h post transfection.

### Affinity measurement

Binding kinetics of the RSPO-derived proteins to LGR5, RNF43, or ZNRF3, and binding of the anti-ASGR1 Fab to human and mouse ASGR1 were determined by BLI using Octet Red 96 (PALL ForteBio, Fremont, CA) instruments at 30 °C, 1,000 rpm with either streptavidin (SA) or anti-hIgG Fc capture (AHC) biosensors. Biotinylated extracellular domain (ECD) of RNF43, ZNRF3 or ASGR1 and Fc portion of human IgG1-fused LGR5-ECD (R&D Systems, Minneapolis, MN) were diluted to 50 nM in the running buffer (PBS, 0.05% Tween-20, 0.5% BSA, pH 7.2) captured to the SA biosensor and the AHC biosensor, respectively, until coupling level reached ~ 1.0 nm. Following capture of ECDs of RNF43, ZNRF3 or LGR5, the biosensors were dipped into wells containing the relevant test molecules at seven different concentrations in running buffer plus a well with only running buffer as a reference channel. K_D_ was determined by steady-state analysis based on the average responses between 140 to 145 s of association phase for binding to LGR5, RNF43 and ZNRF3. For the binding to ASGR, K_D_ was determined by curve model fitting using the *Octet* Red data analysis software (PALL ForteBio, Fremont, CA).

### Cell flow cytometry

HEK293 cells transiently transfected with a plasmid overexpressing *ZNRF3* (GenScript OHu22977), alone or together with *ASGR1*, and were treated for 24 h with RSPO derivative molecules at 10 nM final concentration with or without 10% Wnt3a-conditioned media. Cells were dissociated using Gibco enzyme-free dissociation buffer, washed, and resuspended in FACS buffer (1X PBS with 1% BSA with 0.02% sodium azide). Cells were incubated with 1 nM 18R5 IgG for 1 h. After washing, the cells were incubated with goat anti-human IgG Alexa Fluor 647 (Invitrogen, Carlsbad, CA) for 40 min. Cells were washed with FACS buffer and subjected to multi-channel analysis using a BD Accuri C6 Plus Flow Cytometer (BD Biosciences, San Jose, CA). Data were processed with FlowJo software (FlowJo, Ashland, OR) and fluorescence signals were displayed in histogram plots.

### Western blot analysis of cellular proteins

Western Blot analysis was performed as previously reported^9^, with minor modifications. In brief, HuH7 or HEK293 cells were treated with RSPO mimetics or controls in the presence of 10% Wnt3a conditioned media for 6.5 (HuH-7) or 22 (HEK293) hrs before harvesting for cell lysis. The anti-LRP6, anti-phospho-LRP6 (Ser 1490) and anti-DLV2 primary antibodies were from Cell Signaling Technology (Danvers, MA). Anti-α Tubulin antibody was from Sigma-Aldrich (St Louis, MO). Chemiluminescent signals were detected with X-ray films with various exposure times.

### Quantitative PCR analysis of gene expression

RNA from human cell cultures (HEK293, HuH-7 and A431) and RNA from mouse tissues (liver and small intestine samples) was extracted using the Qiagen RNeasy Micro Kit (Qiagen, Hilden, Germany) and the MagMAX mirVana Total RNA Isolation Kit (ThermoFisher, A27828), respectively. cDNA was produced using the SuperScript IV VILO cDNA Synthesis Kit (ThermoFisher, Waltham, MA). RNA was quantified using TaqMan Fast Advanced Master Mix with the following probes Hs01005019_m1 *ASGR1*, Hs00154160_m1 *ASGR2*, Hs00951083_m1 *TFRC*, Hs01060665_m1, Mm00443610_m1 *Axin2*, Mm01278617_m1 *Ki67*, Mm00431715_m1 *Afp*, Mm00487224_m1 *Cyp1a2*, Mm00491127_m1 *Cyp2e1*, Mm00432359_m1 *Ccnd1*, Mm00552558_m1 *Rnf43*, Mm01191453_m1 *Zrnf3*, Mm01300555_g1 *wnt1*, Mm00470018_m1 *wnt2*, Mm00437336_m1 *wnt3*, Mm01194003_m1 *wnt4*, Mm00437347_m1 *wnt5a*, Mm01183986_m1 *wnt5b*, Mm00437353_m1 *wnt6*, Mm00437356_m1 *wnt7a*, Mm01301717_m1 *wnt7b*, Mm01157914_g1 *wnt8a*, Mm00457102_m1 *wnt9b*, Mm00442104_m1 *wnt10b*, Mm00437327_g1 *wnt11*, Mm00446420_m1 *wnt16*, Mm00507077_m1 *rspo1*, Mm00555790_m1 *rspo2*, Mm01188251_m1 *rspo3*, Mm00615419_m1 *rspo4* probes (ThermoFisher, 4331182). Values were normalized to expression of constitutive *ActinB* RNA using Mm02619580_g1 (ThermoFisher, Waltham, MA).

### Animal

All animal experimentation was in accordance with the criteria of the National Academy of Sciences: Guide for the Care and Use of Laboratory Animals. Protocols for animal experimentation were approved by the Surrozen Institutional Animal Care and Use Committee. Mice were acclimatized a minimum of two days prior to initiating experiments. Mice had unlimited access to purified, laboratory-grade acidified water and were fed ad libitum (2018 Teklad global 18% protein rodent diet). Mice were kept on a 12/12-h light/dark cycle in a 30% to 70% humidity environment and room temperature ranging from 20 to 26 °C.

In a first study, six-week old C57BL/6J male mice were obtained from Jackson Laboratories (Bar Harbor, ME, USA) and were group-housed. Mice were injected intraperitoneally (i.p.) with α-β-gal-IgG, Fc-RSPO2-WT, αGFP-RSPO2-RA or αASGR1-RSPO2-RA at indicated doses (n = 8 per group). 48 h after protein dosing, mice were anesthetized with isoflurane and blood was removed by cardiac puncture. Portions of the left liver lobe and duodenum were collected and either snap-frozen for qPCR analysis or fixed in formalin for 24 h, then transferred to 70% EtOH for immunohistochemistry analysis.

In additional studies, six-week old C57BL/6J male mice were treated with TAA. In study #1 (Fig. 5B,C), TAA was added to drinking water at a concentration of 200 mg/L for twenty weeks to induce liver fibrosis. In addition, during the last six weeks of TAA conditioning, mice were administered with TAA i.p. 3 times weekly. In study #2 (Fig. 5D), TAA was added to drinking water for eighteen weeks. Mice were also administered with 200 mg/kg TAA i.p. 3 times weekly during the last nine weeks of TAA exposure. TAA treatment was discontinued 2 days prior to dosing with recombinant protein, and mice returned to purified, laboratory-grade acidified drinking water. Mice were injected intraperitoneally (i.p.) with recombinant α-β-gal-IgG (10 mg/kg) or Fc-RSPO2-WT (10 mg/kg) twice weekly, or αASGR1-RSPO2-RA-IgG (10 mg/kg) daily. At times indicated, INR was measured using the Roche CoaguChek – XS Plus. At day 0 (right before protein treatment), or 3, 7 or 14 days after beginning dosing, mice were anesthetized with isoflurane and blood was removed by cardiac puncture. A portion of the left liver lobe and duodenum were collected for analysis. Total Bilirubin was measured using a Vet Axcel chemistry analyzer and a total bilirubin reagent (SA1008) (Alfa-Wasserman, West Caldwell, NJ).

### Immunofluorescence and histology

Formalin-fixed and paraffin-embedded liver samples were sectioned and stained with the Ki-67 rat antibody (Invitrogen, Carlsbad, CA), and the HNF-4-alpha rabbit antibody (Abcam, Cambridge, UK). The number of Ki-67 positive nuclei per randomly chosen field (200 × magnification using 20 × objective) were counted using the Leica LAS X software (Leica Microsystems, Wetzlar, Germany). Hematoxylin-eosin was performed according to standard procedures.

## Supplementary information

Supplementary Figures.
