# Supplementary material for: Tissue-targeted R-spondin mimetics for liver regeneration
Source: Sci Rep. 2020 Aug 18;10:13951. doi: 10.1038/s41598-020-70912-3 (PMC7435267; doi:10.1038/s41598-020-70912-3)
Supplement: Supplementary file 1 — Supplementary Figures. [file 41598_2020_70912_MOESM1_ESM.docx]

**Supplementary Figures**

**
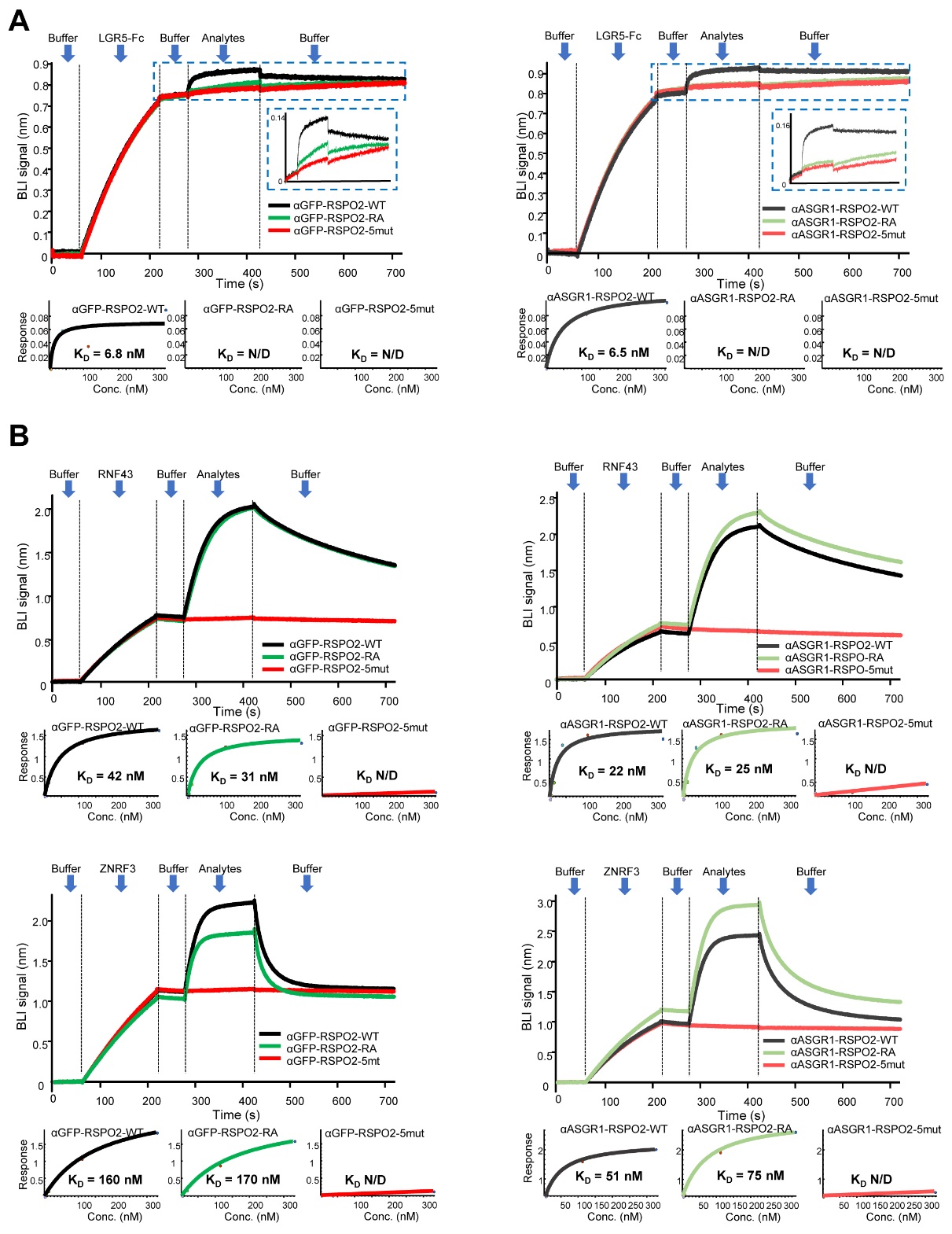
**

**Figure S1.** BLI analysis on LGR5 (A) or ZNRF3/RNF43 (B) binding to the anti-GFP series (*left*) and anti-ASGR1 series (*right*) of fusions. Entire steps of the binding assay with 111 nM of RSPO2 mimetics are shown on top. The binding response composed of association and dissociation phases are separately shown in the box with blue dotted line. The average responses between 140 to 145 seconds of association phase from seven different concentrations of RSPO2 mimetics were analyzed by steady-state analysis to determine K_D_ values (*bottom*). N/D = not determined.


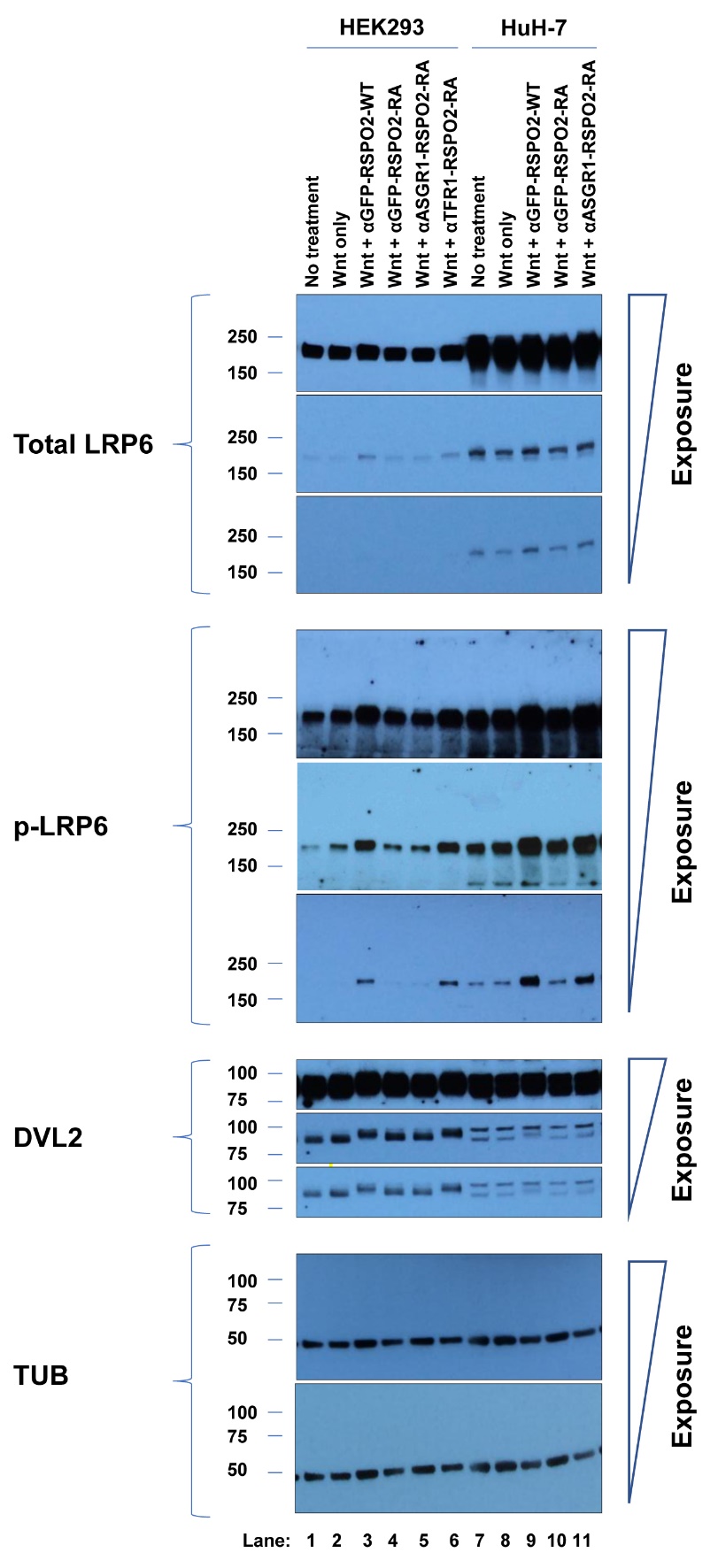


**Figure S2. Uncropped Western Blot images.** Western Blot analysis on LRP6 receptor and DVL2 phosphorylation in HEK293 and HuH-7 cells. Each blot was split into 2~3 pieces each for different antibodies. An area of the X-ray film image that is slightly bigger than the corresponding trimmed membrane was shown. Different exposures were included. Lanes 1-6 correspond to Figure 3B, and lanes 7-11 correspond to Figure 1E.

**
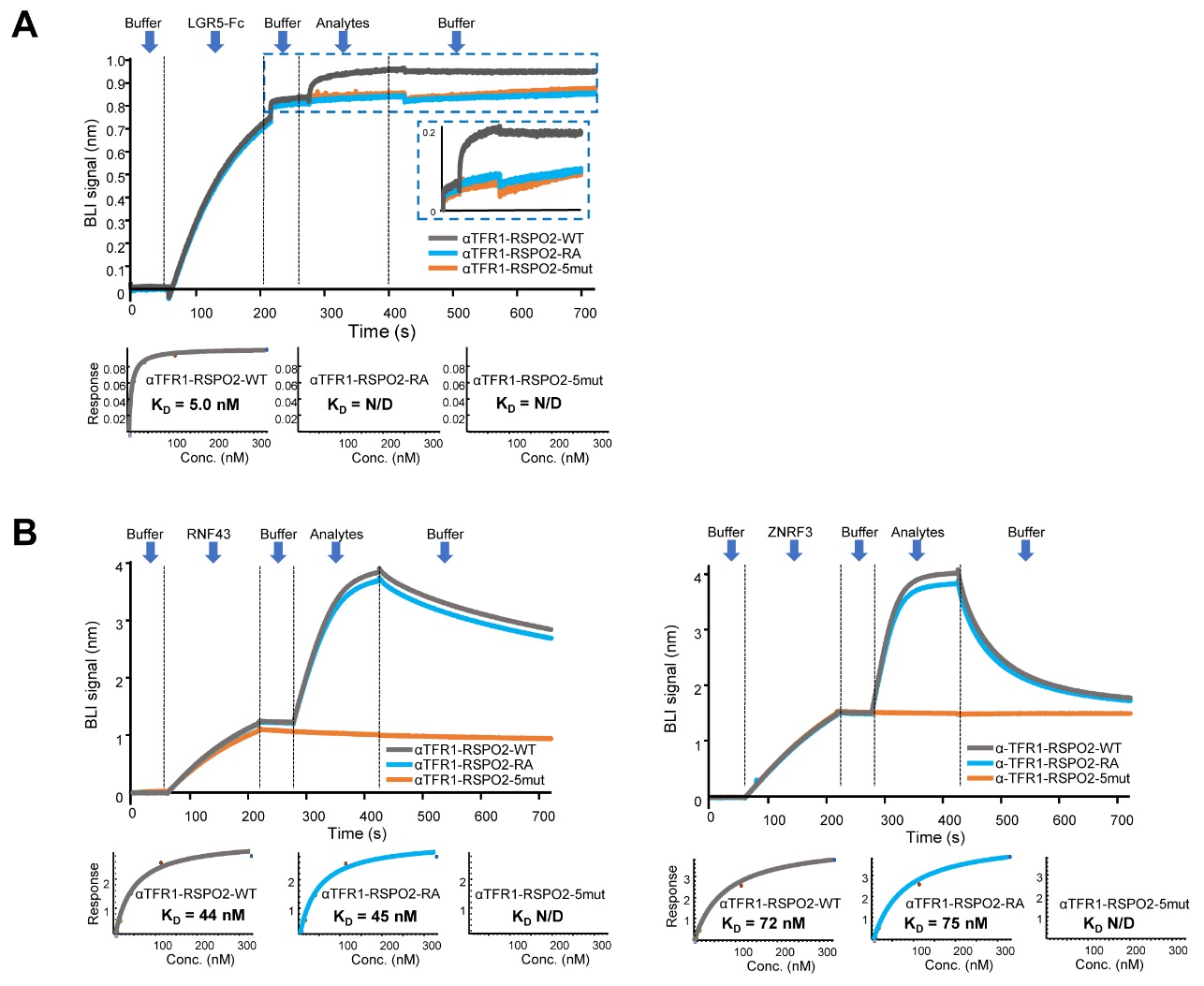
**

**Figure S3.** BLI analysis on LGR5 (A) or ZNRF3/RNF43 (B) binding to the anti-TFR1 series of mimetics. See Figure S1 legends for more details.


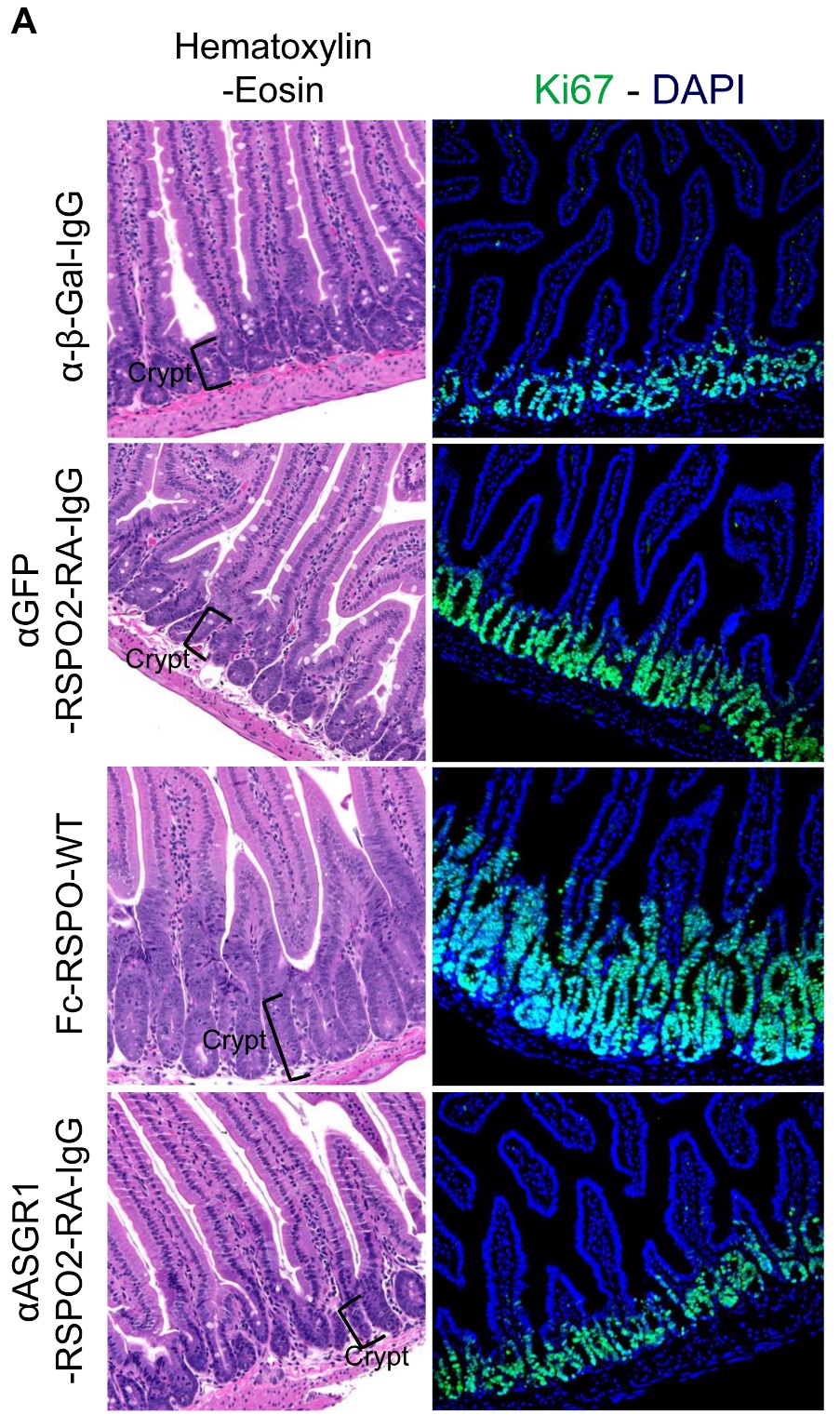


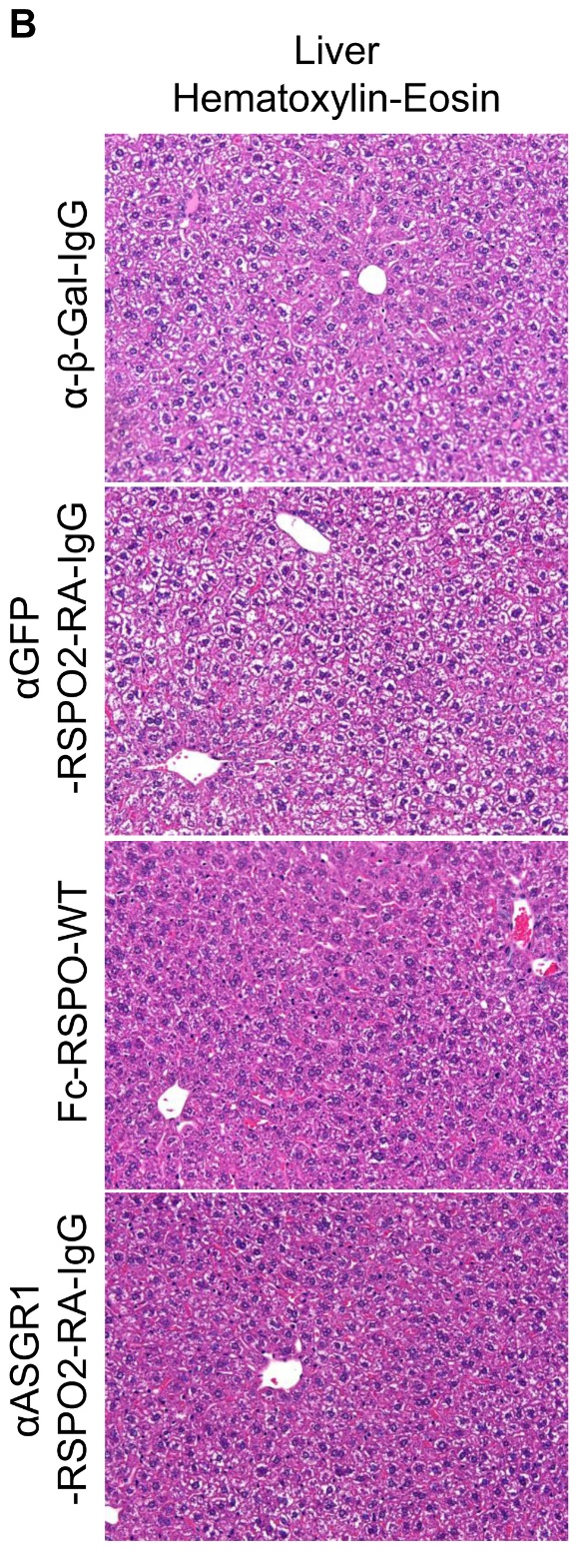


**Figure S4.** *In vivo* tissue-specific enhancement of Wnt signaling. (A) Small intestine histological and immunofluorescence staining of healthy mice in response to a single dose of either Fc-RSPO2-WT, αASGR-RSPO2-RA-IgG, αGFP-RSPO2-RA-IgG or α-β-Gal-IgG. (*left*) Hematoxylin-eosin staining. Bracket indicates crypt height. (*right*) Ki67 antibody staining (green) with DAPI counterstaining (blue) indicates proliferating intestinal cells. (B) Liver histological analysis of healthy mice in response to a single dose of either Fc-RSPO2-WT, αASGR-RSPO2-RA-IgG, αGFP-RSPO2-RA-IgG or α-β-Gal-IgG.

**A.**


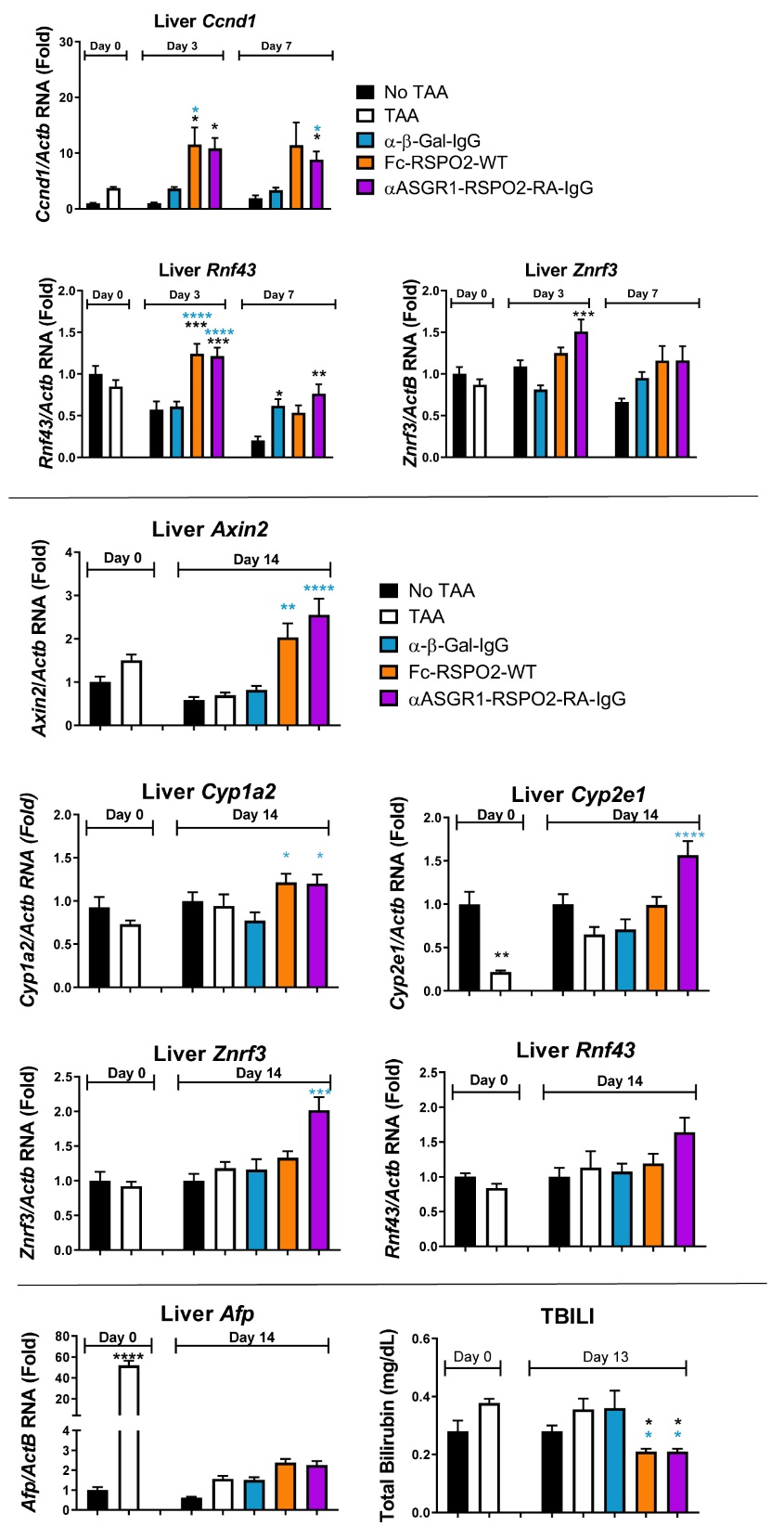


**B.**

**
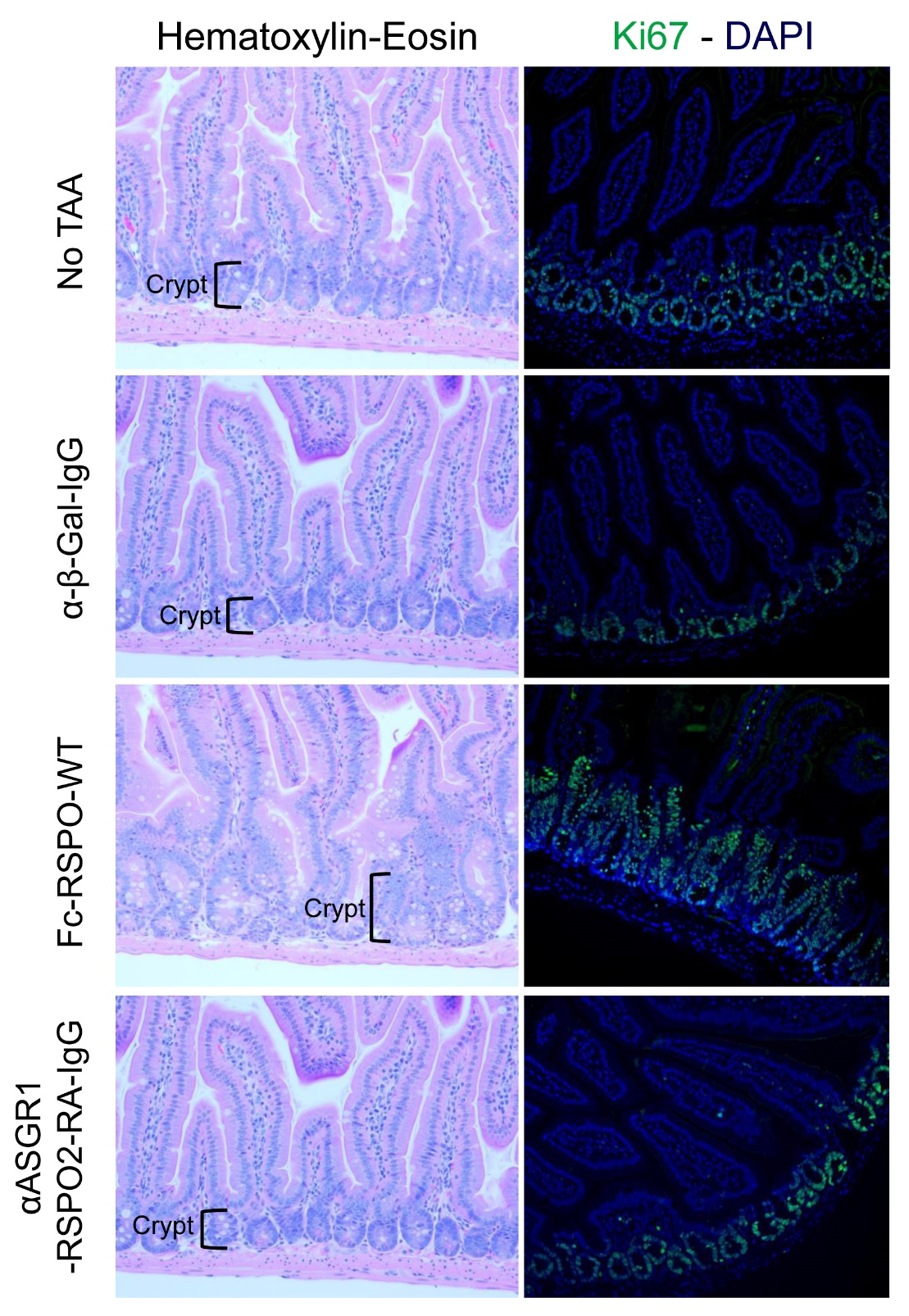
**

**C.**

**
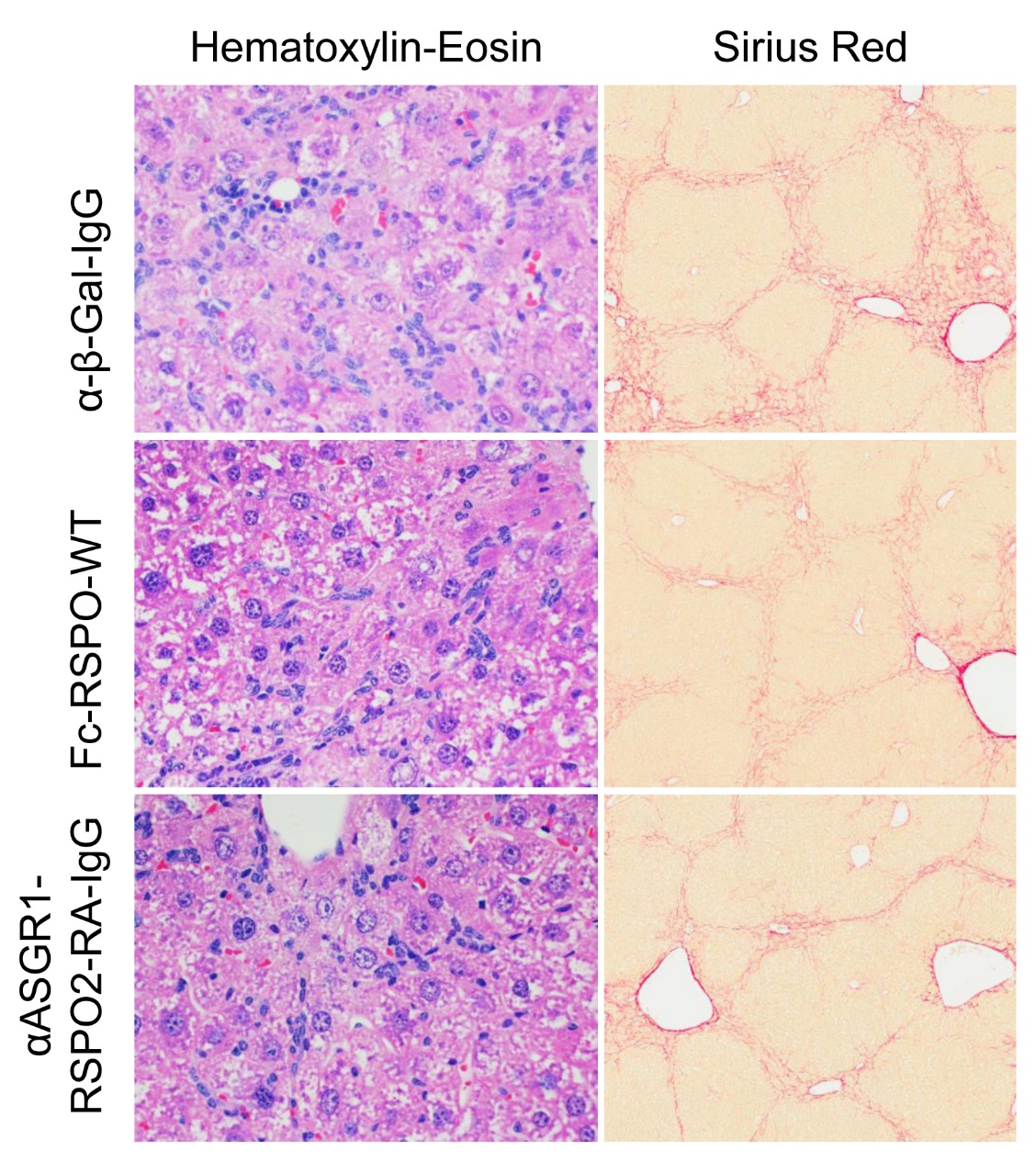
**

**Figure S5.** Effects of liver targeted RSPO mimetic in a TAA mouse model of chronic liver disease. (A) Expression analysis of the Wnt target genes *Axin2*, *Ccnd1*, *Cyp1a2*, *Cyp2e1*, *Znrf3* and *Rnf43*, in livers of TAA-treated mice in response to Fc-RSPO2-WT, αASGR-RSPO2-RA-IgG or α-β-Gal-IgG. Samples from mice not exposed to TAA (No TAA) were included as controls. Tissues were collected at baseline (day 0) and at day 3 or day 7 or at day 14 after start of dosing of test articles. (n=10 mice per group). (B) Small intestine histological and immunofluorescence staining of TAA-treated mice in response to 14 days of treatment with either Fc-RSPO2-WT, αASGR-RSPO2-RA-IgG or α-β-Gal-IgG. (*left*) Hematoxylin-eosin staining. Bracket indicates crypt height. (*right*) Ki67 antibody staining (green) with DAPI counterstaining (blue) indicates proliferating intestinal cells. (C) Liver histological analysis of TAA-treated mice in response to 14 days of treatment with either Fc-RSPO2-WT, αASGR-RSPO2-RA-IgG or α-β-Gal-IgG. (*left*) Hematoxylin-eosin staining shows the presence of oval cells and monocytes in all groups. (*right*) Picrosirius red staining shows the presence of fibrosis in all groups.
